# Fertility-Sparing Treatment in Young Women with Atypical Endometrial Hyperplasia and Low-Grade Endometrial Cancer: A Tertiary Center Experience

**DOI:** 10.5935/1518-0557.20200037

**Published:** 2020

**Authors:** Sumaya Shikeli, Vaidyanathan Gowri, Thuria Al Rawahi

**Affiliations:** 1 Oman Medical Specialty Board, Muscat, Oman; 2 Sultan Qaboos University Hospital, Muscat, Oman; 3 Royal hospital Oman, Muscat, Oman

**Keywords:** Endometrial hyperplasia, endometrial cancer, fertility, progestogens

## Abstract

**Objectives:**

To evaluate the oncologic and reproductive outcomes of patients with atypical endometrial hyperplasia and low grade stage 1 endometrial cancer who received medical treatment.

**Methods:**

We carried out a retrospective study on all patients aged 20-42 years with complex, atypical endometrial hyperplasia, and low-grade stage 1 endometrial cancer, who wished to preserve fertility and were treated at the Royal Hospital. We held this study between January 2006 and December 2016. The patients received oral megestrol acetate with or without a levonorgestrel intrauterine system. We assessed their response to progestin treatment in terms of treatment duration, time to response, pregnancy, time of surgery, and oncological outcome. We performed the statistical analysis using the SPSS 20.0 software.

**Results:**

Twenty patients met the inclusion criteria, and among them 90% had complete remission. Among these 90%, 55% had complete remission within six months of treatment. The recurrence rate was recorded in 11 patients (55%) and it was more frequent in obese patients with body mass index (BMI) ≥30 (*p*=0.001), who had complete response in > 6 months of hormonal treatment. About 15% of the patients required hysterectomy, and 12 (60%) patients conceived after full treatment.

**Conclusions:**

Fertility-sparing treatment of atypical endometrial hyperplasia and grade 1 stage 1 endometrial cancer in reproductive-age women is feasible. However, obese patients (BMI ≥ 30) had a higher recurrence rate.

## INTRODUCTION

Endometrial cancer (EC) may develop from precursor lesions, mainly endometrial hyperplasia (EH). Twenty-nine percent of untreated atypical hyperplasia may progress to cancer (Zaino *et al*., 2006). Both of them (EC and EH) histologically share the same risk factors of unopposed estrogen therapy, obesity, diabetes, nulliparity, early menarche, and late menopause, which are all related to excess estrogen, compared to progesterone (Aoki, 2014; Soliman *et al*., 2005). The definitive treatment for atypical hyperplasia and endometrial cancer in young women is surgery, which consists of hysterectomy with or without removing the ovaries (Soliman *et al*., 2005; Kaaks *et al*., 2002). However, most of the times this type of treatment is not a choice for young women, particularly the nulliparous ones, wishing to preserve fertility.

Endometrial hyperplasia and endometrial cancer are estrogen-dependent, and hence progestins are often used to induce their regression. When arising in young women, endometrial cancer usually presents with favorable prognostic features that is, a focal, well-differentiated lesion, with minimal or absent myometrial invasion. For these women, hormone therapy (generally progestins) has been used as a conservative treatment in many countries. (Inoue *et al*., 2016).

Growing evidence shows that fertility-preserving treatment can be used for young women with atypical hyperplasia and early-stage, low-grade endometrial carcinoma. However, there is no local published data on long-term outcomes and prognostic factors.

The goal of this study was to evaluate the oncologic and reproductive outcomes of reproductive-age patients with atypical endometrial hyperplasia and low grade endometrial cancer who were managed conservatively, using medical treatment between 2006 to 2016 in the Royal hospital, Sultanate of Oman. This study was conducted because there is no national published data or studies that investigated the outcomes of medical treatment of atypical endometrial hyperplasia and low grade endometrial cancer in this population.

## MATERIALS AND METHODS

We ran a retrospective study using data from patients with atypical hyperplasia and early endometrioid endometrial cancer in a reproductive-age group of women treated with oral progestins, levonorgestrel-releasing intrauterine system (LNG-IUS) or combination as fertility-sparing treatment between January 1, 2006, and December 31, 2016, Royal Hospital, Oman. The inclusion criteria for selecting the patients were: age of 20-45 years who desired to preserve their fertility and had stage 1A, grade 1 endometriod endometrial cancer, diagnosed by histopathology and on the basis of the staging system from the International Federation of Gynecology and Obstetrics. The study was approved by the Center of Studies and Research of the hospital. The body mass index (BMI) was calculated as weight in kilograms divided by the square of height in meters), and the cut-off point was set at 30 according to the current international classiﬁcation of the World Health Organization for obesity.

### Treatment and evaluation of response

The progestin therapy consisted of either oral megestrol acetate (MA) at a dose of 160 mg/day or levonorgestrel-releasing intrauterine system (LNG-IUS) or a combination of megestrol acetate and levonorgestrel-releasing intrauterine system (MA + LNG-IUS).

Response to progestin treatment was assessed histologically using specimens obtained via hysteroscopic biopsies or out-patient biopsies with a pipelle curette. The histological evaluation was performed every 6 monthly. A complete remission was deﬁned as the absence of hyperplasia or malignancy. Once patients achieved a complete remission, a maintenance therapy was given for 3-6 months in case of atypical hyperplasia, and 12 months in cases of endometrial cancer. The recurrence rate was deﬁned as the time (in months), from full treatment to the date of relapse.

### Statistical Analysis

The frequency distributions of the baseline characteristics were compared using the Fisher exact tests when the expected frequency was less than ﬁve, and the Pearson χ2 test was used when the expected frequency was higher than five. We used the SPSS 20.0 software to perform the statistical analysis. *p*<0.05 was considered signiﬁcant.

## RESULTS

A total of 20 patients met the inclusion criteria and were included in the study. Out of these twenty patients, 19 had atypical hyperplasia and one had endometrial cancer. Their median age at diagnosis was 31 years. The BMI was higher than 30kg/m^2^ in 60% of the women with a mean BMI of 33.1kg/m^2^. Eleven patients (55%) were diagnosed during fertility investigations. Nineteen patients (95%) complained of abnormal menstruation and 70% of the patients were diagnosed with polycystic ovarian syndrome (Table 1).

**Table 1:** Characteristics of the patients treated with progestins

Patient characteristics		Atypical hyperplasia (n=19)	Endometrial cancer (n=1)
Age	<35 ≥35	14 5	1
Body Mass Index	<30 ≥30	8 11	1
Parity	Nullip parous	10 9	1
Irregular periods	Yes	18	1
Polycystic ovarian syndrome	Yes	13	1
Diabetes mellitus	Yes	9	1

Of the 20 patients, 11 (55%) received MA alone. The LNG-IUS was placed in one (5%) patient and 8 (40%) received a combination of MA and LNG-IUS (Table 2). The mean duration of progestin treatment for all patients was 6.35 months.

**Table 2:** Treatment options of progestin for the patients

Treatment	Atypical hyperplasia	Endometrial cancer
Megestrol Acetate	11 (57.89%)	0 (0%)
Mirena Coil	1 (5.27%)	0 (0%)
Megestrol+ Mirena	7 (36.84%)	1 (100%)

Eighteen women had complete disease remission, and two (10%) did not have full remission. Of these two, one had atypical hyperplasia and one had endometrial cancer. Of these 18 women, 55% patients had complete remission upon 6 months from starting treatment. There was no relation between complete response to the treatment and age, BMI, previous pregnancy, PCOS, initial endometrium histopathology, or progestin treatment type (Table 3).

**Table 3:** Prognostic factors of complete response to progestin treatment (n=18).

Variables	Complete Remission	*p* value
Age <35 ≥ 35	14/15 4/5	0.447
Body Mass Index < 30 ≥ 30	8/9 10/11	1.000
Previous Pregnancies Yes No	8/10 10/10	0.47
Irregular periods Yes No	18/19 0/1	0.1
Diabetes Mellitus Yes No	9/10 9/10	1.0
Polycystic Ovarian Syndrome Yes No	12/14 6/6	1.0
Histopathology Atypical Endometrial cancer	18/19 0/1	0.52
Progestin Type Megestrol Acetate Mirena Coil Megesterol+Mirena	11/11 1/1 6/8	0.343

The mean follow-up period after complete remission was 8 months (range 6-24). Of the 20, 55% of the patients had a relapse (Table 4). The median time to recurrence was 18 months. Ten out of 11 patients received a second cycle of progestin, and one patient progressed to endometrial cancer, but she refused surgery. A total of 3 patients (15%) underwent hysterectomy. Those who experienced recurrence had a BMI ≥ 30 (*p*=0.001) and had treatment for over 6 months till complete response (*p*=0.010) (Table 4).

**Table 4:** Relevant factors associated with recurrence after complete response to progestin treatment (n=11)

Variables	Recurrence	*p* value
Age <35 ≥35	10/15 (66.7%) 1/5 (20%)	0.127
Body Mass Index < 30 ≥30	1/9 (11.1%) 10/11 (90.9%)	0.001
Diabetes Mellitus Yes No	5/10 (50%) 6/10 (60%)	1.000
Polycystic Ovarian Syndrome Yes No	9/14 (64.3%) 2/6 (33.3%)	0.336
Histopathology Atypical Endometrial cancer	10/19 (52.63%) 1/1 (100%)	0.304
Progestin Type Megestrol Acetate Mirena Coil Megesterol+Mirena	5/11 (45.6%) 0/1 (0%) 6/8 (75%)	0.299
Time to complete remission 6 months >6 months	3/11 (27.3%) 8/9 (88.9%)	0.010
Pregnancies after treatment Yes No	6/12 (50%) 5/8 (62.5%)	0.670

Twelve (60%) patients conceived after full treatment, with a total of 12 pregnancies. (10 live-births and 2 miscarriages). Ten pregnancies (71.4%) were achieved thanks to assisted reproductive technology and ovulation induction therapies 

The average time to conception was 7.4 months. Five women were submitted to *in vitro* fertilization and eight women had ovulation induction. However, the recurrence rate of hyperplasia was almost equal in both groups (about 40%). Four women had cesarean section but there was no pregnancy with placenta previa or accrete.

## DISCUSSION

There have been many reports on conservative managements on atypical endometrial hyperplasia and low grade, early stage of endometrioid endometrial cancer. This study also has indicated that progestin treatment (Megestrol acetate and LNG-IUS) is one method of treatment for patients with atypical hyperplasia or grade 1 endometrial cancer, with high complete remission rate (90%). Ushijima *et al*. (2007) reported in a prospective multicentre study a very high response rate both in atypical complex hyperplasia and in endometrial cancer (100 and 87%, respectively. In another report by Yamazawa *et al*. (2007) found a response rate of 50-100%.

Progestin treatment is not the curative therapy for atypical hyperplasia and endometrial cancer, and there is risk of disease progression with the therapy with recurrence rate of 55% in present study. Patients with endometrial cancer have been reported to have a lower response rate than do those with atypical hyperplasia (55% *vs.* 82%; Ushijima *et al*., 2007) and (48% *vs.* 66%; Gunderson *et al*., 2012). So patients, especially with endometrial cancer, should understand the possibility of disease progression during progestin treatment and should not choose medical management if they desire to conceive.

The results also suggest that obese patients (BMI ≥30) have an increased risk of recurrence. Many studies reported that obesity was the main factor that associated with failure to achieve complete response to the treatment. Chen *et al*. (2016) found that a BMI of at least 30 was the only signiﬁcant factor associated with failure to achieve a complete remission and also was associated with a higher risk of recurrence.

Patients with atypical hyperplasia, or endometrial cancer had history of infertility, anovulation and abnormal menstrual bleeding, PCOS and obesity, which all can reduce their chance of pregnancy even after full treatment. Therefore, responders to the treatment should be referred to a fertility treatment. In the present study, the pregnancy rate was 60% which is higher than the previously reported rates of 23% (Ushijima *et al*., 2007), 52% (Gunderson *et al*., 2012) and 32% (Koskas *et al*., 2014).

The limitations of this study it is retrospective character and the fact that it was carried out in one single center.

## CONCLUSION

Fertility-sparing strategies for treatment of endometrial hyperplasia and endometrial cancer in reproductive-age women are effective. Patients with high BMI (BMI ≥ 30) had high recurrence rates. Therefore, the attending physician should also focus on weight reduction for these patients.

Progestin therapy is not the definitive treatment for atypical hyperplasia and endometrial cancer, and only specific patients should be given the choice of this treatment after extensive counseling about the risks.
